# Analysis of Hyperexpanded T Cell Clones in SARS ‐ CoV ‐2 Vaccine‐Associated Liver Injury by Spatial Proteomics and Transcriptomics

**DOI:** 10.1111/liv.70172

**Published:** 2025-06-16

**Authors:** Sarp Uzun, Asmita Pant, Ewelina Bartoszek, Paul Gueguen, Stefan Frei, Hélène Heusler, Ilaria Arborelli, Carl Philipp Zinner, Neşe Karadağ Soylu, Benedetta Terziroli Beretta‐Piccoli, Cumali Efe, Matthias S. Matter

**Affiliations:** ^1^ Institute of Pathology University Hospital of Basel, University of Basel Basel Switzerland; ^2^ Microscopy Core Facility, Department of Biomedicine University of Basel Basel Switzerland; ^3^ Functional Genomics Center Zurich, ETH Zurich/University of Zurich Zurich Switzerland; ^4^ Department of Pathology University of Health Sciences, Adana City Education and Research Hospital Adana Turkey; ^5^ Servizio di Gastroenterologia Ed Epatologia Ente Ospedaliero Cantonale, Ospedale Civico Lugano Switzerland; ^6^ Faculty of Medical Biosciences Università della Svizzera Italiana Lugano Switzerland; ^7^ MowatLabs, Faculty of Life Sciences & Medicine King's College London, King's College Hospital London UK; ^8^ Department of Gastroenterology Harran University Hospital Şanlıurfa Turkey

**Keywords:** autoimmune hepatitis, COVID‐19, SARS‐CoV‐2, vaccination, vaccine‐induced liver injury

## Abstract

**Background and Aims:**

SARS‐CoV‐2 vaccine‐associated liver injury (SVALI) is a rare event and its pathophysiology remains unclear. Previous studies have found an oligoclonal CD8+ T cell infiltrate and SARS‐CoV‐2 spike antigen‐specific T cells in the liver of patients with SVALI. Therefore, we aimed to characterise the immune infiltrate in a liver explant from a patient with severe SVALI.

**Methods:**

T cell receptor sequencing, a novel combined multiplex immunofluorescence (mIF)‐RNA in situ hybridisation (RISH) approach, and single cell spatial transcriptomics with the Xenium in situ platform were used to identify, track and characterise specific T cell clones in this liver sample.

**Results:**

T cell repertoire analysis revealed hyperexpanded clones with CDR3 sequences similar to previously identified SARS‐CoV‐2 spike antigen‐specific T cells. The hyperexpanded clones were localised throughout the whole liver, but the concentration was higher at the portal interface. Many hyperexpanded T cells expressed cytotoxic granzymes A, B and K, but also tissue‐resident markers such as CXCR6, CD69 and KLRB1.

**Conclusions:**

Spatial proteomics and spatial transcriptomics techniques allowed the localisation and characterisation of hyperexpanded CD8+ T cell clones at single cell level. They exhibited cytotoxic and tissue‐resident memory properties, suggesting their involvement in the pathogenesis of SVALI.

**Abbreviations**
AIH: autoimmune hepatitis
CDR3: complementarity‐determining region 3
DEG: differentially expressed genes
FFPE: formalin‐fixed and paraffin‐embedded
mIF: multiplex immunofluorescence
PBC: primary biliary cholangitis
RISH: RNA in situ hybridisation
SARS‐CoV‐2: severe acute respiratory syndrome coronavirus 2
SVALI: sars‐CoV‐2 vaccine‐associated liver injury
TCR: t cell receptor
T_RM_
: tissue‐resident memory T cells

**Summary**

- In a patient with severe, treatment‐resistant SVALI, the liver showed a strong immune response dominated by CD8+ T cells, with several T cell clones hyperexpanded.
- The hyperexpanded T cell clones were already present in a previous liver biopsy and shared receptor sequences (TCRβ‐CDR3) with spike‐specific CD8+ T cells, suggesting that they may target the spike protein.
- The hyperexpanded T cell clones were spread throughout the liver but were particularly concentrated at the portal interface close to hepatocytes.
- The hyperexpanded T cell clones showed features of resident memory T cells (TRM) and carried markers of cytotoxicity, indicating their potential to directly damage liver cells.

## 1 Introduction

SARS‐CoV‐2 vaccine‐associated liver injury (SVALI) is a rare event that usually presents with distinct molecular, morphologic and serologic features [1, 2, 3]. It is characterised by a CD8 T cell dominant immune infiltrate consisting of SARS‐CoV‐2 spike‐specific CD8 T cells [4, 5]. We recently analysed the transcriptome and immune infiltrate of SVALI by comparing a cohort of six SVALI patients and nine AIH patients. Our study identified a strong CD8+ T cell infiltration with an oligoclonal immune repertoire [4]. Despite these previous observations, the exact contribution of spike‐specific CD8 T cells to SVALI, their phenotype and their spatial distribution is poorly understood. Therefore, we analysed the liver explant from a single patient who underwent liver transplantation due to liver failure after his second dose of Pfizer BioNTech COVID‐19 vaccine (BNT162b2) [6]. Using T cell receptor (TCR) sequencing, we identified hyperexpanded T cell clones that shared complementarity‐determining region 3 (CDR3) similarity with a previously described SARS‐CoV‐2 spike‐specific T cell clone [7, 8, 9]. Therefore, our aim in this study was to characterise and localise these hyperexpanded CD8+ T cell clones on a single cell level. To this end, we developed a novel combination of multiplex immunofluorescence (mIF) with mRNA in situ hybridisation (RISH) and performed spatial transcriptomics using the Xenium in situ platform. Our study suggests the involvement of SARS‐CoV‐2 spike‐like cytotoxic T cells with resident phenotype in the disease pathogenesis while we also present novel approaches to localise and phenotype T cell clones in situ.

## 2 Materials & Methods

The materials and methods can be found in the Data S1.

## 3 Results

**FIGURE 1:**
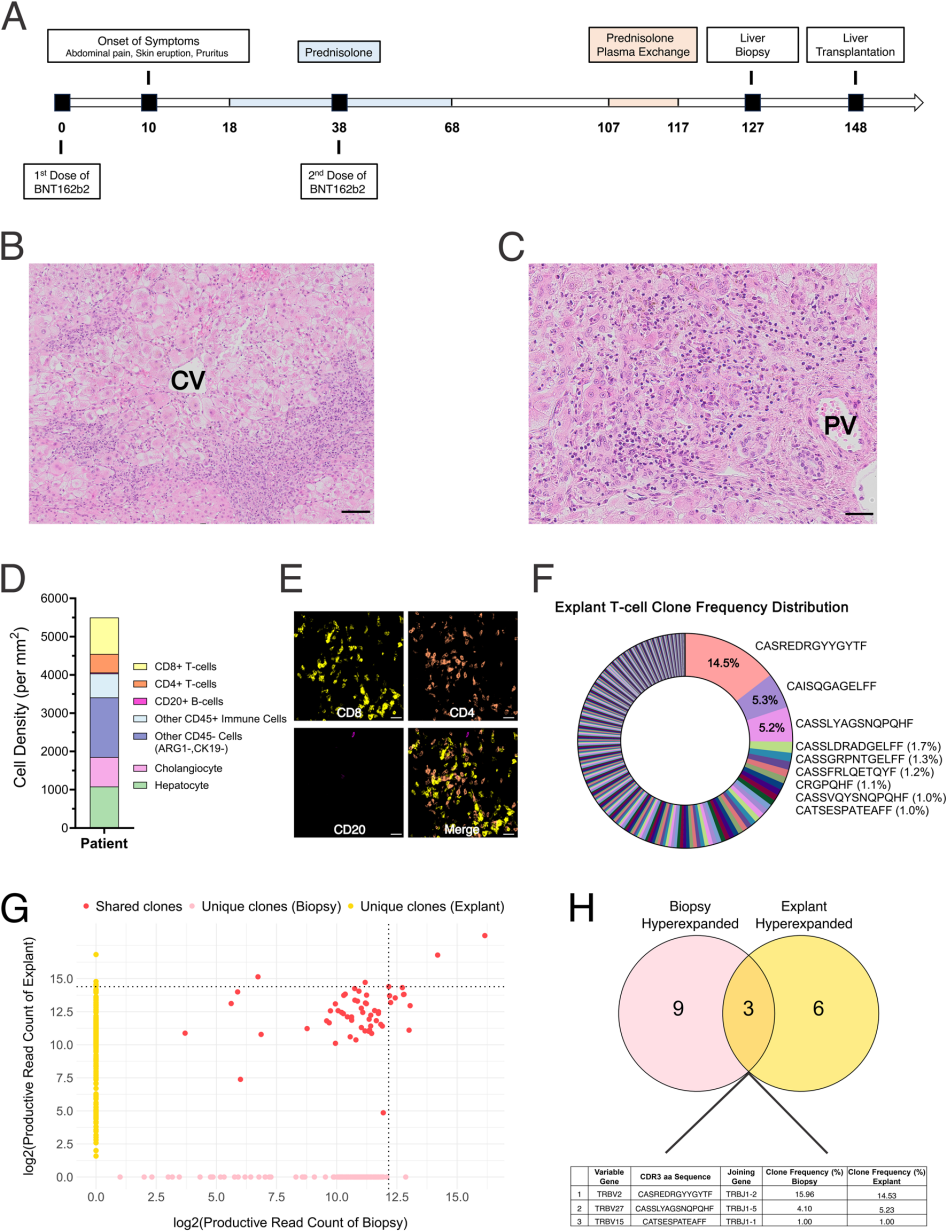
Analysis of immune infiltrate in the liver explant of the SVALI patient. (A) Clinical course of the patient. (B), (C) Haematoxylin and eosin (H&E) staining of liver explant showing lobular hepatitis (B) and interface hepatitis (C). Scale bar = 100 μm (B) and 50 μm (C). (D) Cell density measured by mIF. (E) Representative image for CD8, CD4, CD20 and merge. Scale bar = 20 μm. (F) T cell clone frequency and CDR3 sequence of the hyperexpanded clones in the explant. (G) Scatter plot of the unique and shared T cell clones in the liver biopsy and explant. Dotted lines indicate the threshold for hyperexpanded clones. (H) Hyperexpanded T cell clones shared between the previous liver biopsy and the explant.

We analysed the liver explant from a 53‐year‐old male patient previously reported as one of the most severe cases of SVALI [6]. Briefly, after a first dose of BNT162b2 vaccine, the patient developed mild abdominal pain, erythematous skin rash and elevated liver enzymes, which were successfully treated with steroids. Six weeks after the first vaccination, the patient received a second dose of BNT162b2 vaccine, while still on steroids. He again developed similar symptoms but worsened over the following weeks and became refractory to steroid treatment and plasma exchange. A liver biopsy performed 89 days after the second vaccine dose showed severe portal and lobular inflammation. Because the clinical course worsened, the patient underwent liver transplantation 110 days after the second vaccine dose (Figure 1A). He had an uneventful postoperative course, and 2 years after the transplantation, the patient is in good health.

Histology of the liver explant showed severe portal and lobular inflammation with interphase hepatitis consisting of a lymphocytic infiltrate with a few plasma cells and neutrophilic granulocytes (Figure 1B,C). Few focal lytic necrosis or apoptosis of hepatocytes were found, but no confluent necrosis. Fibrosis was mild. We performed mIF using the Phenocycler‐Fusion system to profile immune cells. PhenoGraph‐based unsupervised cell clustering [10] (Figure S1A) revealed a strong CD8 positive T cell infiltrate accounting for 17.1% of all analysed cells, a less pronounced CD4 positive T cell (8.7%) and a weak CD20 positive B cell (0.5%) infiltrate (Figures 1D,E). Besides hepatocytes (19.4%) and cholangiocytes (13.8%), we also detected clusters with other CD45+ immune cells (11.2%) and CD45‐ cells other than hepatocytes and cholangiocytes (28%) (Figure 1D).

TCRβ‐CDR3 sequencing on RNA isolated from the liver explant revealed 508 T cell clones, of which nine constituted more than 1% of all TCRβ‐CDR3 sequences and were therefore considered hyperexpanded (Figure 1F and Figure S1B). The most expanded T cell clone represented 14.5% of the T cell repertoire, while the next most expanded clones represented 5.3% and 5.2% of the T cell repertoire. We wanted to know whether T cell clones persisted in the liver as the disease progressed. Therefore, we searched for T cell clones shared between the explant and the liver biopsy that was performed 21 days prior to transplantation. TCRβ‐CDR3 sequencing of the liver biopsy revealed 189 T cell clones, which was lower than in the explant and most likely explained by the significantly lower tissue volume of the liver biopsy. 12 T cell clones constituted more than 1% of all TCRβ‐CDR3 sequences in the liver biopsy and were therefore hyperexpanded (Table S1 and Figure S1C). Interestingly, 59 T cell clones were shared between the liver biopsy and the explant (Figure 1G). In addition, 10 of the 12 hyperexpanded T cell clones from the liver biopsy were also present in the liver explant, of which 3 clones, termed clone 1–3, were still hyperexpanded in the explant (Figure 1H). This included the most expanded T cell clone (clone 1), described above, which comprised 16% of the T cell repertoire in the liver biopsy and 14.5% in the explant. We further used a curated database of TCR‐CDR3 sequences to understand whether CDR3 sequences matched with TCRs of known antigen specificities [11]. All three hyperexpanded T cell clones shared between the liver biopsy and explant matched with T cell clones previously identified as specific to the SARS‐CoV‐2 spike epitope ‘YLQPRTFLL’ [7, 8, 9] (Figure S1C), suggesting that these clones recognise the mRNA‐derived spike antigen.

**FIGURE 2:**
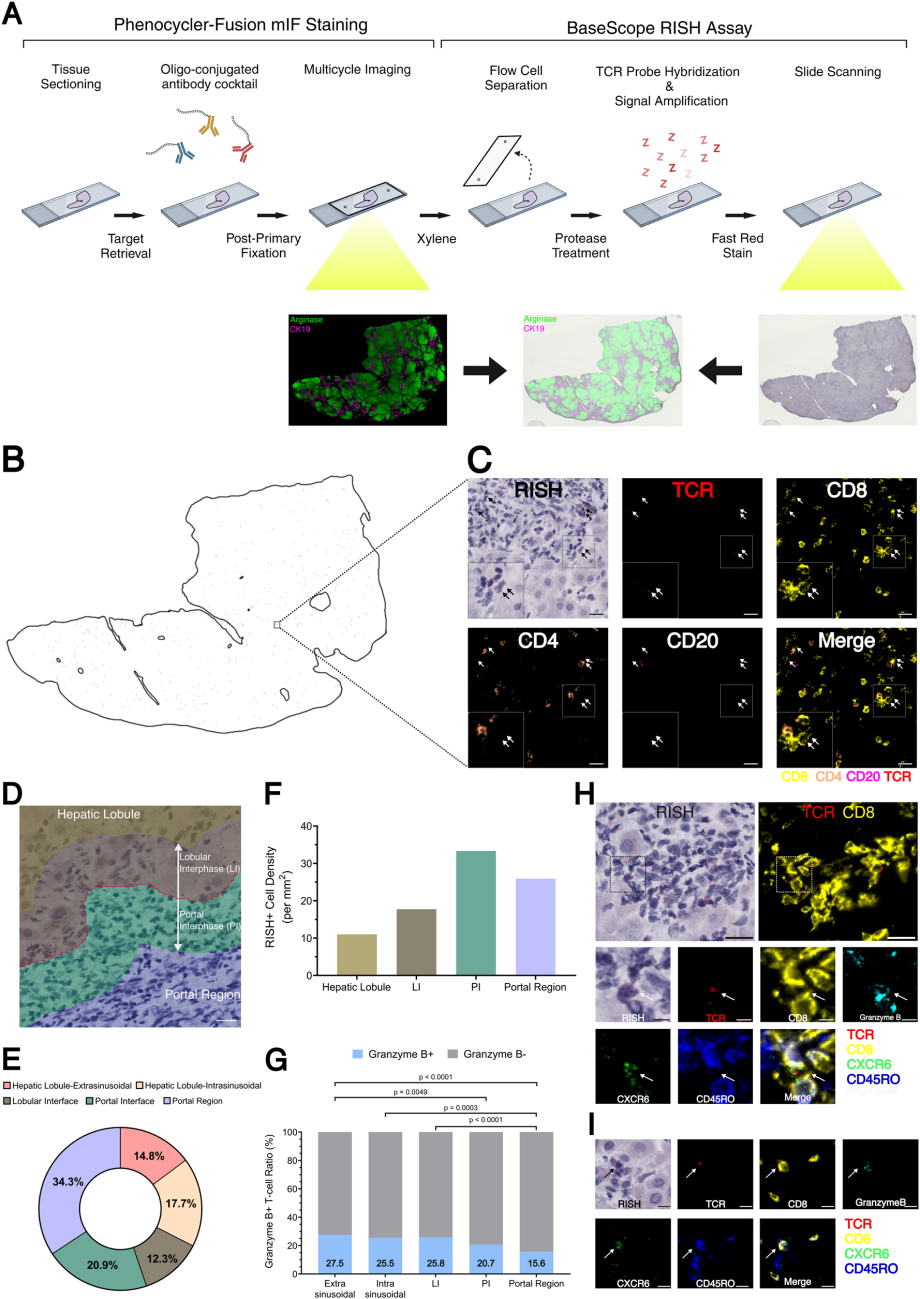
Localisation and phenotyping of clone 1 with combined mIF and RISH.(A) Workflow of combined mIF and RISH (BioRender, 2024). (B) Each black dot in the liver drawing represents a RISH+ T cell. (C) mIF images from an area enlarged from (B). Arrows show RISH+ and therefore TCR+ cells (red). CD8: Yellow, CD4: Orange, CD20: Magenta. Scale bar = 20 μm. (D) Division of the liver into different zones by using a pixel classifier trained on Arginase staining. Scale bar = 20 μm. (E) Percentage of RISH+ cells in the different zones: Hepatic lobule extrasinusoidal and intrasinusoidal, lobular interface, portal interface, portal region. (F) Density of RISH+ CD8+ T cells and (G) Frequency of RISH+ CD8+ Granzyme B+ T cells in the different liver zones. (H) Phenotypic characterisation of RISH+ CD8+ cells. Scale bar = 20 μm. Inset: The arrow shows RISH+ CD8+ Granzyme B+ CXCR6+ CD45RO‐ cell. Scale bar = 5 μm. (I) Identification of RISH+ CD8+ Granzyme B+ CXCR6+ CD45RO+ cells (arrow). Scale bar = 10 μm.

Given that the top T cell clone represented 14.5% of the T cell repertoire in the liver explant, we wanted to take the opportunity to investigate the phenotype and localisation of this T cell clone in more detail. Therefore, we developed an assay where we first performed mIF using different cell lineage and state markers (Figure 2A and Table S2). Afterwards, we performed RISH on the same tissue section by using a custom‐designed probe targeting the CDR3 mRNA sequence of clone 1 (Figure 2A). Finally, we co‐registered both images, which allowed us to localise and characterise the phenotype of T cell clone 1 with T cell specific lineage and state markers at single cell resolution. We detected 2585 RISH‐positive cells, which were evenly distributed in a liver tissue section of 144 mm^2^ (18 cells/mm^2^) (Figure 2B). All RISH‐positive cells were CD8+ T cells (Figure 2C), which were confirmed by a dual RISH and immunohistochemistry assay (Figure S2A). Interestingly, the frequency of RISH‐positive cells among all CD8+ T cells was 1.9%. Neither CD4+ nor CD20+ cells were found to be positive for RISH (Figure 2C).

To analyse the immune cell distribution, we generated tissue masks with a pixel classifier [12] that was trained on arginase positive hepatocytes to divide the liver into five zones: (1) the classical portal tract, (2) an area extending 50 μm from the limiting plate into the portal tract called the portal interface (PI), (3) an area extending 50 μm into the hepatic lobule called the lobular interface (LI), and finally, the hepatic lobule, reduced by 50 μm, was divided into (4) an intrasinusoidal compartment, where cells are located within the blood circulation and (5) an extrasinusoidal compartment, where cells are located within the hepatic lobule (Figures 2D). Out of the 2585 RISH‐positive CD8+ cells, 34.3% were found in the portal region, 20.9% in the PI, 12.3% in the LI, 17.7% intrasinusoidal and 14.8% extrasinusoidal (Figures 2E). RISH‐positive CD8+ T cells showed the highest density in the PI zone (33.3/mm^2^) and the lowest in the hepatic lobule (11/mm^2^), combined for intra‐ and extrasinusoidal (Figures 2F). As expected from the type of liver inflammation, which was purely hepatocytic, only 10 (0.04%) RISH‐positive CD8 T cells were found to infiltrate CK19+ bile ducts (Figure S2B). Further, we wanted to know how many of RISH‐positive CD8 T cells had an effector phenotype which was defined by Granzyme B positivity. 21.8% of the RISH‐positive CD8 T cells showed an effector phenotype which was significantly higher than among RISH‐negative CD8 T cells (13.2%) (*p* < 0.0001) (Figure S2C). The frequency of RISH+ Granzyme B+ cells within the total RISH+ T cells was highest within the extrasinusoidal compartment of the hepatic lobule (27.5%), followed by the lobular interface (25.8%), the intrasinusoidal compartment (25.5%), the portal interface (20.7%) and lowest in the portal region (15.6%) (Figure 2G), indicating that there was a trend of RISH+ effector T cells being localised within the hepatic lobules or interphase. RISH+ CD8 T cells showed various phenotypes and a small portion of cells also expressed the chemokine receptor CXCR6 (7.2%) which is a marker for liver‐resident CD8 T cells (Figure 2H), and CD45RO (4.3%), a memory T cell marker (Figure 2I). The distribution of CXCR6+ RISH+ and CD45RO+ RISH+ cells was similar across the different zones of the liver, but it was less pronounced in the intrasinusoidal compartment (Figure S2D and S2E).

**FIGURE 3:**
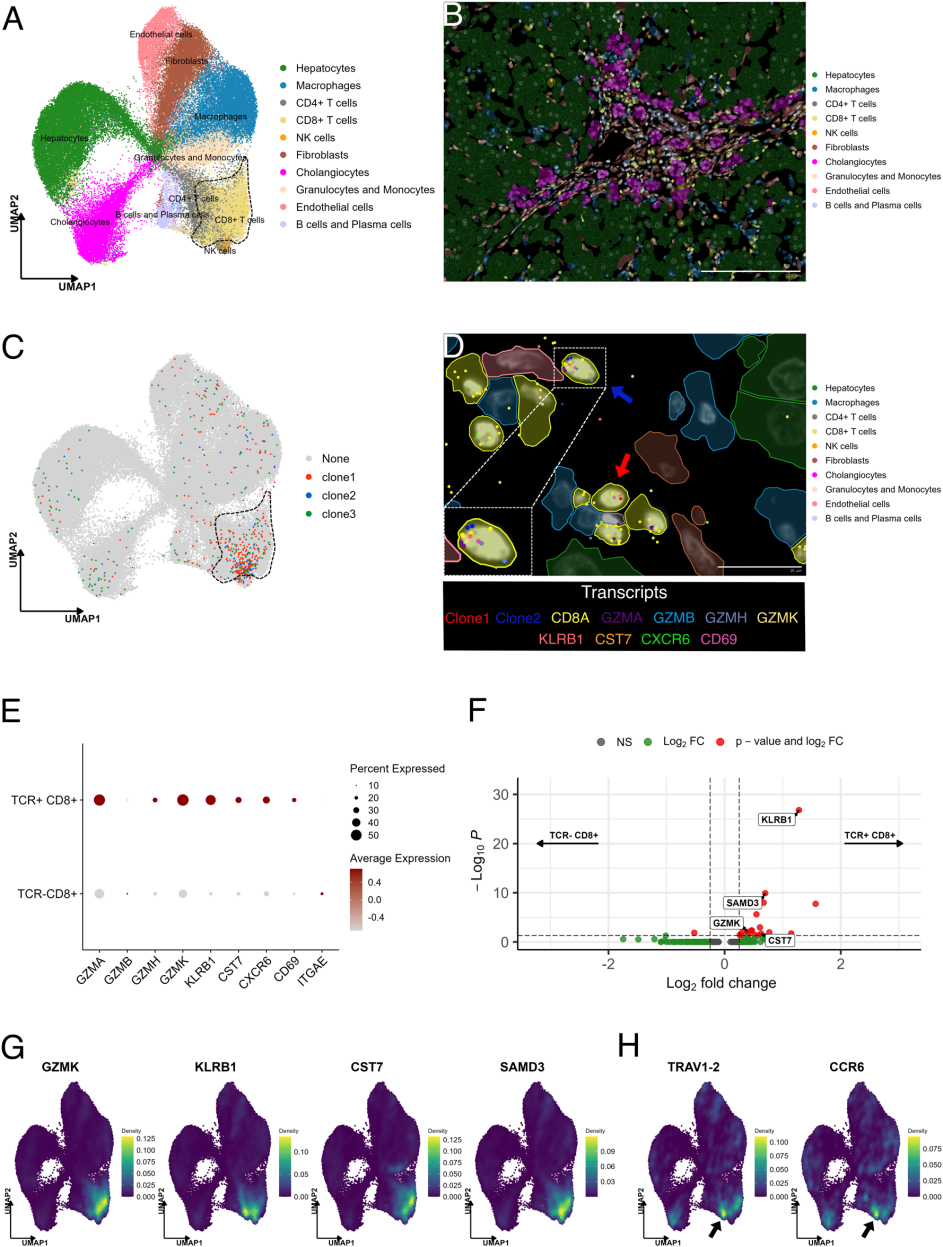
Localisation and phenotyping of clone1, clone2 and clone3 with Xenium in situ. (A) Dimension reduction of the Xenium in situ data yielded a UMAP projection with 10 clusters. Each point represents a cell, and the colours show annotated cell types. The dotted region indicates the CD8 T cell cluster. (B) Mapping of the cell type annotation mask onto the explant tissue shows the portal region in the centre and surrounded by the hepatic lobule. Scale bar = 200 μm. (C) UMAP projection of Xenium in situ data shows hyperexpanded T cell clones (clone 1, clone 2 and clone 3). Each point represents a cell, and the colours show different cells with at least 1 TCR transcript. The dotted region indicates the CD8 T cell cluster. (D) A portal region in the liver explant. Cell annotation masks indicate different cell types. Each dot represents an mRNA transcript detected by Xenium in situ, with transcripts colour‐coded as shown below the figure. Red arrow: Clone 1; blue arrow: Clone 2. Scale bar = 20 μm. Inset: Clone 2 with residency and cytotoxicity genes it expresses. (E) Average expression of cytotoxicity and residency markers in TCR (clone 1/clone 2/clone 3 combined) + CD8+ and TCR‐ CD8+. Dot size indicates the percentage of cells expressing the indicated marker and the colour bar shows the scaled average expression of the marker. (F) Differentially expressed genes (DEGs) between TCR+ CD8+ T cells and TCR‐ CD8+ T cells (log_2_FC > 0.25, p_adj_ < 0.05). (G) Gene expression density distribution on a UMAP projection for the selected DEGs: *KLRB1*, *SAMD3*, *GZMK* and *CST7*. (H) Gene expression density distribution on a UMAP projection for the MAIT cell markers *TRAV1‐2* and *CCR6*. Black arrows indicate the CD8+ T cell cluster with a higher expression of *TRAV1‐2* and *CCR6*.

The main limitations of the combined RISH and mIF assay are that RISH can only identify one specific T cell clone at a time and the number of markers for mIF is limited. We therefore performed a spatial transcriptomics experiment using the Xenium in situ platform [13]. We designed custom probes that target the CDR3 sequences of the three hyperexpanded clones (clone 1–3) which are shared between liver biopsy and liver explant (Figure S1D and Table S3). In addition, we added custom‐designed probes for selected genes such as *HEPN1*, *GLUL* for hepatocyte identification and *KRT7*, *KRT19* for cholangiocyte identification, to a pre‐designed probe panel (Human Immuno‐Oncology Panel) (Table S4). Next, we performed a spatial transcriptomics experiment with the combined probe panel containing 449 genes and 3 CDR3 probes. The region of interest in the liver explant was selected based on high clone 1 infiltration from the previous RISH analysis. Cell segmentation was performed with standard Xenium segmentation algorithm [13]. As a result, 78.271 segmented cells were assigned to 10 main cell lineages, including parenchymal and non‐parenchymal cells of the liver (Figure 3A). Although only a selected region of the whole explant tissue was used for Xenium spatial transcriptomics, cell type annotation based on gene expression profiles revealed a similar cell composition to that previously obtained with mIF (Figure 1D). Within total cells, CD8+ T cells constituted 10.3%, CD4+ T cells 5.9%, B cells and plasma cells 2.4%, hepatocytes 26% and cholangiocytes 12.3% (Figure 3A,B, Figure S3A). 45% of TCR transcripts from clone 1–3 were identified within CD8+ T cells and the rest of the transcripts were assigned to various other cell types (Figure 3C). This is most likely explained by CD8+ T cells being in close proximity to neighbouring cells, causing the TCR signal to be detected on the latter—a phenomenon known as signal bleed‐through [14]. Alternatively, CD8+ T cells could be taken up by other cells through phagocytosis or emperipolesis [15, 16]. We restricted T cell clone analysis to CD8+ T cells with at least one transcript from clone 1, 2 or 3. A total of 268 TCR+ CD8+ cells was detected, accounting for 3.3% of all CD8+ T cells. Within the CD8+ cells, the frequencies of clone 1, clone 2 and clone 3 were 2.35%, 0.54% and 0.42%, respectively. TCR+ CD8+ T cells were distributed throughout the whole liver explant, but were enriched in the portal region, in close proximity to hepatocytes (Figure 3D). In comparison to TCR‐ CD8+ T cells and similar to the combined RISH/mIF data, TCR+ CD8+ T cells showed more pronounced expression of genes associated with cytotoxicity (e.g., *GZMA*, *GZMH*, *GZMK*, *CST7*) (Figure 3E). Only *GZMB* expression levels were low compared to mIF staining, most likely due to the accumulation of granzyme B within cytoplasmic granules, while transcriptional activity was low [17]. Likewise, TCR+ CD8+ T cells strongly expressed tissue residency markers such as *CXCR6* (34.7%), *CD69* (23.1%) and *KLRB1* (CD161) (47.4%), which suggested that many hyperexpanded T cell clones display tissue‐resident memory (T_RM_) T cell phenotype (Figure 3E). In contrast, *ITGAE* (CD103) showed little expression. The differential gene expression analysis showed significantly higher expression of tissue residency marker *KLRB1* (log2FC: 1.28), together with other genes‐related to cytotoxicity such as *SAMD3* (log2FC: 0.70), *GZMK* (log2FC: 0.39) and *CST7* (log2FC: 0.63) in TCR+ CD8+ T cells compared to TCR‐ CD8+ T cells (Figure 3F,G Table S5). Interestingly, the three TCR clones (clone 1–3) showed a similar gene expression profile of tissue residency markers and cytotoxicity (Figure S3C). As expected from the combined mIF/RISH assay, TCR+ CD8+ T cells showing the expression of cytotoxicity markers were also identified in the sinusoidal hepatic blood circulation Figure S3D, Figure S3E).

Innate‐like T cell populations called mucosal‐associated invariant T (MAIT) cells are abundant in the liver and strongly express *KLRB1* (CD161), in addition to the invariant TCR α‐variable 1–2 (*TRAV1‐2*), *CCR6* and usually *CD8* [18]. Because the hyperexpanded CD8+ T cell clones showed a strong expression of *KLRB1*, we wondered whether they also co‐expressed MAIT cell markers. However, TCR+ CD8+ T cells did not show co‐expression of *KLRB1* and *TRAV1‐2*, which indicated that TCR+ CD8+ T cells are CD8+ CD161+ non‐MAIT cells (Figure 3H).

In conclusion, spatial transcriptomics performed with the Xenium platform supported the combined mIF/RISH analysis and revealed that hyperexpanded T cell clones in the liver of a patient with severe, treatment‐refractory SVALI showed a T_RM_ phenotype with strong expression of cytotoxicity markers.

## 4 Discussion

SARS‐CoV‐2 mRNA vaccines are generally well tolerated and are only rarely associated with serious adverse events [1, 19]. However, as mRNA vaccines continue to be used, it is important to understand their mechanism of adverse events. In this study, the liver explant from a critically ill patient showed a CD8‐dominated immune infiltrate with multiple hyperexpanded clones. These clones showed similarity to SARS‐CoV‐2 spike‐specific T cell clones, directly linking them to prior vaccination [20]. In addition, many of these clones persisted in the liver as the patient's condition worsened, suggesting their involvement in disease progression.

Among all T cell clones identified in the liver explant, the top clone accounted for 14.5% of the entire T cell repertoire, as measured by TCR sequencing on isolated RNA. Hyperexpansion of T cell clones has been described after vaccination or infection and is only surpassed by lymphomas, where these values can reach almost 100% [21, 22]. This high expansion of a specific T cell clone representation allowed us to further characterise it by using a combined assay of mIF and RISH. The feasibility of combined protein and nucleic acid imaging techniques has already been tested in different multiplex imaging platforms, such as imaging mass cytometry and CO‐Detection by indexing (CODEX) [23, 24, 25]. To our knowledge, our study is the first to combine mIF and RISH to detect a specific T cell clone. In addition, we performed another novel approach by using a high resolution spatial transcriptomics platform [13] to track and phenotype hyperexpanded persistent T cell clones. Both techniques allowed us to localise and characterise antigen‐specific T cells in the liver on a single cell level. Only CD8 T cells were positive for the CDR3 mRNA sequence of clone 1 by RISH, representing 1.9% of the total CD8 T cell population. This proportion was lower than expected based on TCR sequencing results but is most likely explained by the fact that TCR sequencing was conducted on isolated total RNA, whereas RISH staining was performed on a tissue section at single cell level. Indeed, spatial transcriptomics on the liver explant revealed a frequency of 2.35% within CD8+ T cells for clone 1, confirming RISH data. CD8+ RISH+ T cells were distributed throughout the whole liver, but the highest density was observed at the portal interface and they were almost completely absent from the bile ducts. Similarly, activated (Granzyme B+) CD8+ RISH+ T cells were mainly found at the interphase but also within hepatic lobules, consistent with the elevated liver enzymes. Interestingly, mIF revealed that some RISH+ CD8+ T cells expressed the liver‐resident T cell marker CXCR6, which was shown to be expressed in tissue‐resident memory T cells (T_RM_) [26], a subset of T cells previously described to be important in SVALI [5]. The frequency of CD8+ CXCR6+ T cells measured by mIF was lower than previously shown, possibly due to differences in sensitivity between flow cytometry‐based staining and mIF staining. Indeed, our results from the single cell transcriptomics data further confirmed that hyperexpanded T cell clones frequently expressed tissue‐resident markers *CXCR6* (34.7%), *CD69* (23.1%) and *KLRB1* (47.4%). *KLRB1* was significantly higher expressed in hyperexpanded CD8 T cell clones compared to TCR negative CD8 T cells. In addition, hyperexpanded clones strongly expressed cytotoxic markers, suggesting that T cell clones with T_RM_ phenotype may be cytotoxic in SVALI. A cytotoxic function has been attributed to T_RM_ cells in several liver diseases, including AIH and primary biliary cholangitis (PBC) [26]. However, T_RM_ cells were CD103 positive in AIH and PBC [27, 28]. Whereas T_RM_ cells in our study and in a recent study on liver transplant rejection showed low CD103 expression [29].

The primary limitation of our study is that spatial T cell tracking was performed in only a single patient, using one liver explant tissue block. Nevertheless, our results, derived from multiple complementary techniques, are consistent with our previous findings and those of others, supporting the potential generalisability of our observations to a broader patient population with severe SVALI. In addition, although we have provided some insight into the cells involved in SVALI, we do not fully understand the mechanisms by which this occurs. Hyperexpanded clones may target hepatocytes presenting spike antigens on MHC‐I molecules, as evidence indicates that spike antigen‐encoding mRNA is expressed within the hepatocyte cytoplasm in SVALI patients [30]. Alternatively, they may cross‐react with human peptides due to molecular mimicry, given the substantial peptide sharing between the SARS‐CoV‐2 spike glycoprotein and human proteins [31]. However, it is still unclear why only a small number of patients are predilected to disease formation [32]. Recently, genetic variants of the MHC I pathway proteins ERAP1 and ERAP2 were found to be overrepresented in an SVALI cohort, suggesting a role for alterations in intracellular antigen processing in disease pathogenesis and potentially linking genetic susceptibility to disease development [33]. Indeed, our patient had the GG phenotype of ERAP2 rs1363907 that was shown to be more prevalent in high causality SVALI cases (Figure S4).

In conclusion, the integration of TCR sequencing, combined mIF/RISH and spatial transcriptomics revealed that a patient with severe, treatment‐refractory SVALI exhibited clonal hyperexpansion of T cells bearing CDR3 sequences similar to those previously identified as spike‐specific. These hyperexpanded T cells were already present in a previous liver biopsy and demonstrated a cytotoxic T_RM_ cell phenotype. These findings unify and extend observations from two prior studies: one reporting an oligoclonal T cell repertoire in a SVALI cohort, and another identifying spike‐specific T_RM_ cells in a SVALI patient [4, 5]. Importantly, we show for the first time that these hyperexpanded T cells are localised specifically at the portal interface, in close proximity to hepatocytes, suggesting a key role in hepatocellular injury and disease pathogenesis.

## Author Contributions

Conceptualization: S.U., M.S.M.; Data Collection and Formal Analysis: S.U., A.P., E.B., P.G., S.F., H.H., I.A., M.S.M.; Funding acquisition: M.S.M.; Project administration: N.K.S., B.T.B.‐P., C.E., M.S.M.; Writing – original draft: S.U., M.S.M.; Writing – review and editing: All authors.

## Ethics Statement

The study was approved by the ethics commission of Northern Switzerland (EKNZ; study ID: 2020–00969) and the local ethical review board of Harran University Hospital (HRU/2021.17.29). Written informed consent was obtained from the patient included in the study, and the study conformed to the ethical guidelines of the Declaration of Helsinki.

## Conflicts of Interest

M.S.M. has served as a consultant for ThermoFisher, Merck, GlaxoSmithKline, Janssen‐Cilag, Roche, Novartis and received speaker's honorary from Incyte Biosciences and Astellas. Otherwise, the authors have no conflicts of interest to declare.

## Supporting information


**Figure S1.** T cell immune repertoire of liver explant and biopsy of SARS‐CoV‐2 vaccine‐associated liver injury (SVALI) patient
**Figure S2.** In situ localisation and phenotyping of clone #1 in liver explant
**Figure S3.** In situ localisation and phenotyping of clone 1, 2 and 3 in liver explant with Xenium in situ
**Figure S4.** The Sanger sequencing of the genomic region covering ERAP2 rs1363907
**Table S1.** T cell immune repertoire of liver explant and liver biopsy samples
**Table S2.** Reagents used in Phenocycler‐Fusion Experiment
**Table S3.** Probe sequences for hyperexpanded T cell clones
**Table S4.** Add‐on custom gene panel
**Table S5.** Differentially expressed genes between TCR+ CD8+ cells and TCR‐ CD8+ cells

Data S1.

## Data Availability

T cell receptor sequencing raw data can be provided upon request. The csv files of TCR sequencing data containing T cell clone details can be found in Table S1.
