# Supplementary material for: Analysis of Hyperexpanded T Cell Clones in SARS ‐ CoV ‐2 Vaccine‐Associated Liver Injury by Spatial Proteomics and Transcriptomics
Source: Liver Int. 2025 Jun 16;45(7):e70172. doi: 10.1111/liv.70172 (PMC12169079; doi:10.1111/liv.70172)
Supplement: Supplementary file 1 — Figure S1. T cell immune repertoire of liver explant and biopsy of SARS‐CoV‐2 vaccine‐associated liver injury (SVALI) patient Figure S2. In situ localisation and phenotyping of clone #1 in liver explant Figure S3. In situ localisation and phenotyping of clone 1, 2 and 3 in liver explant with Xenium in situ Figure S4. The Sanger sequencing of the genomic region covering ERAP2 rs1363907 Table S1. T cell immune repertoire of liver explant and liver biopsy samples Table S2. Reagents used in Phenocycler‐Fusion Experiment Table S3. Probe sequences for hyperexpanded T cell clones Table S4. Add‐on custom gene panel Table S5. Differentially expressed genes between TCR+ CD8+ cells and TCR‐ CD8+ cells [file LIV-45-0-s002.docx]

**Analysis of hyperexpanded T cell clones in SARS-CoV-2 vaccine-associated liver injury by spatial proteomics and transcriptomics**

Sarp Uzun^1^, Asmita Pant^1^, Ewelina Bartoszek^2^, Paul Gueguen^3^, Stephan Frei^1^, Hélène Heusler^1^, Ilaria Arborelli^1^, Carl Philipp Zinner^1^, Neşe Karadağ Soylu^4^, Benedetta Terziroli Beretta-Piccoli^5,6,7^, Cumali Efe^8^, Matthias S. Matter^1^

Table of Content

Supplementary materials and methods……………………………………… 2

Fig. S1…………………………………………………………………………… 12

Fig. S2…………………………………………………………………………… 14

Fig. S3…………………………………………………………………………… 16

Fig. S4…………………………………………………………………………… 18

Supplementary Table 1………………………………………………………… 20

Supplementary Table 2………………………………………………………… 21

Supplementary Table 3………………………………………………………… 22

Supplementary Table 4………………………………………………………… 23

Supplementary Table 5………………………………………………………… 25

References……………………………………………………………………… 26

**Supplementary Materials and Methods**

**Patient Information and Morphologic Evaluation**

The clinical course of the development of liver injury after vaccination has been described in detail in a previously published case report [1]. Biopsy and explant specimens were fixed in 10% buffered formalin and embedded in paraffin. 2 μm sections were cut from the liver explant tissue block and stained with hematoxylin and eosin. The morphology of the biopsy specimen was reviewed in a previously published study [2].

The study was approved by the ethics commission of Northern Switzerland (EKNZ; study ID: 2020-00969) and local ethical review board of Harran University Hospital (HRU/2021.17.29). Written informed consent was obtained from the patient included in the study and the study conformed to the ethical guidelines of the Declaration of Helsinki.

**Total Nucleic Acid Isolation from Formalin Fixed Paraffin-Embedded (FFPE) Tissue Blocks**

20 μm thick sections were cut from liver biopsy and liver explant FFPE tissue blocks. Total RNA was extracted from FFPE tissue sections using the AllPrep DNA/RNA FFPE Kit (Qiagen, 80234) according to the vendor's instructions. Final RNA concentrations were measured using the Qubit RNA HS Assay Kit (ThermoFisher, Q32852). RNA quality control was performed with High Sensitivity RNA Screen Type assay (Agilent, 5067-5579) on the 4200 TapeStation System (Agilent) and both total RNA samples contained at least 50% of the RNA fragments with >200 nucleotides in size (DV200).

**Next Generation Sequencing of T cell Receptor β Complementarity Determining Region 3 (TCRβ-CDR3)**

cDNAs were synthesized from total FFPE RNA using the SuperScript VILO cDNA Synthesis Kit (Invitrogen, 11754250). Maximum RNA input was loaded for cDNA synthesis to capture a large pool of T cell clones. Next-generation sequencing (NGS) libraries were prepared using the Oncomine TCR Beta-SR RNA Assay (ThermoFisher, A39359) according to the manufacturer’s instructions. Amplified and barcode-ligated libraries were purified using AMPure XP Reagent (Beckman Coulter, A63880) and quantified with the Ion Universal Library Quantitation Kit (ThermoFisher, A26217). The library pool was prepared by combining equal volumes of libraries at 50 pmol/L concentration and loaded into the Ion 550™ Chip (ThermoFisher, A34537). Libraries were sequenced on an Ion GeneStudio S5 Prime Sequencer (ThermoFisher).

**TCR Sequencing Data Analysis**

Read alignment to the International ImMunoGeneTics (IMGT) database and removal of low quality and off-target reads were performed using Ion Reporter Software (version 5.20) Oncomine TCR Beta-SR w1.4 RNA workflow (ThermoFisher). Identified T-cell clones and immune repertoire diversity metrics (richness, Shannon diversity, normalized Shannon diversity [evenness]) were obtained in csv files from the software (**Supplementary Table 1**). Clonality was calculated as 1-evenness. T cell clones with a frequency greater than 1% were defined as hyperexpanded [3]. Shared T cell clones were defined as T cells with the same variable-joining gene and 100% CDR3 amino acid sequence match. Shared clones were visualized using R packages ggplot2 and ggVenn ^4^. T cell clone V-gene distribution was visualized with spectratyping plots that were generated by Ion Reporter Software (version 5.20).

The VDJdb web browser was used to search for T cell clones with similar CDR3 amino acid sequence to the hyperexpanded shared clones in order to predict antigen specificity [5]. The maximum Levenshtein distance was set to two which is defined as minimum number of edits (substitutions, insertions, and deletions) necessary to transform one CDR3 sequence into another. Epitope species other than SARS-CoV-2 were filtered out.

**RNA In situ Hybridization and Dual RNA In situ Hybridization-Immunohistochemistry with BaseScope Assay**

A 1ZZ in situ hybridization probe targeting CDR3 sequence with flanking framework regions (5'-CCATGTACTTCTGTGCCAGCAGAGAAGACAGGGGATACTATGGC-3') of the hyperexpanded T cell clone1 was custom designed by Advanced Cell Diagnostics (ACD, Design ID: NPR-0044742, Cat. No: 712111). The assay was performed according to the manufacturer’s standard protocol of RNA in situ hybridization (RISH) and dual RNA in situ hybridization/immunohistochemistry (dual RISH/IHC) using the BaseScope v2 RED Assay (ACD, Cat. No: 322910) and the RNA-Protein Co-detection Ancillary Kit (ACD, Cat. No: 323180). Main modifications for liver tissue include 20 minutes of target retrieval with co-detection target retrieval solution and 30 minutes of protease IV treatment. Anti-CD8a monoclonal antibody (1:4000, Cat. No: 66868-1-Ig, clone: 1G2B10, ProteinTech) was used for the immunostaining and developed with an avidin/biotin-based peroxidase system (VECTASTAIN Elite ABC-HRP Kit, PK-6100). The experiment was performed on sequentially cut 4 µm sections of liver explant tissue with the target probe, a positive control probe (PPIB) and negative control probe (DapB). Images were captured using an Olympus BX43 microscope at 40X magnification. The experiment was not carried out on the liver biopsy sample because there was too little residual tissue.

**Combined Multiplex Immunofluorescence (mIF) Staining with Phenocycler-Fusion System and RNA In situ Hybridization with BaseScope Assay**

A 5-µm thick whole tissue section was obtained from the liver explant. The section was stained according to the standard protocol of the Phenocycler-Fusion system. Briefly, the section was deparaffinized in a dry oven at 60^o^C overnight and for 10 minutes in xylene. For heat-induced epitope retrieval, tissue sections were incubated in AR9 buffer (Akoya Biosciences, AR90001KT) for 20 minutes at 98^o^C under normal pressure in a laboratory-type microwave (Milestone, Histopro Rapid Microwave Histoprocessor). The section was incubated with the primary antibody cocktail for 3 hours at room temperature and fixed with 1.6% PFA for 10 minutes, 100% cold methanol for 5 minutes, and Phenocycler fixative reagent for 20 minutes. The stained section was stored in the storage buffer at 4^o^C until the flow cell assembly. The image was acquired with the PhenoCycler-Fusion 2.0 Instrument. The phenocycler reagents and antibodies are shared in **Supplementary** **Table 2**. Following mIF staining, the sample slide-flow cell assembly was separated after overnight incubation in xylene. Then, the standard protocol of BaseScope RISH assay was performed as described above without the target retrieval step. The stained slide was scanned with VENTANA DP 200 slide scanner with 40X objective (Roche).

As a control, we performed RISH in the absence of prior mIF on a consecutive liver tissue section as described in the section “RNA In situ Hybridization and Dual RNA In situ Hybridization-Immunohistochemistry with BaseScope Assay” and detected 3224 RISH-positive cells in 140 mm^2^ of tissue (23 cells/mm^2^) (**data not shown**). Therefore, the combined protocol with multiple rounds of washing required for mIF only moderately reduced the number of RISH-positive cells.

**Data Analysis of combined mIF and RISH Staining**

The acquired mIF staining scan from Phenocycler-Fusion in qptiff format and the RISH scan from the slide scanner as tiff format were opened in QuPath [6]. Both image modalities were then registered using Warpy QuPath extension [7]. The RISH image was used as a reference image, and the mIF image was scaled up 2 times from its original pixel size (0.25µm/pix and 0.50µm/pix, respectively). Images were first manually aligned and final registration was performed based on image intensity with affine transformation and precision of 1µm. mIF stainings were validated by visual inspection and by comparison with the Human Protein Atlas [8]. Antibodies with low signal to background ratio were not included in the further analysis. A pixel classifier was trained to create a liver explant mask. Cell segmentation was performed on nuclear channel DAPI using StarDist2D [9] plugin within QuPath with the following parameters: probability threshold: 0.6, pixel size: 0.5 and cell expansion: 0.5 using the model dsb2018_heavy_augment.pb. The mean cell intensities were then exported from QuPath, cluster identification was performed in a web-based software OmiQ using a phenograph clustering [10] with k=60 based on the following markers: Arginase, CK19, CD45, CD3, CD8, CD4 and CD20. For cluster validation, cells were colour coded according to their cluster ID and superimposed on a multichannel scan for visual inspection. 34 clusters were generated, and each was visually inspected in QuPath. Clusters with similar expression patterns were merged. Four clusters with mixed cell types were reclustered and merged again. The final number of clusters was 7.

To separate hematoxylin and RISH+ staining on RISH scan, colour deconvolution was performed in QuPath and RISH+ cells were visually inspected. An intensity threshold above the background was used to determine the positivity for phenotyping markers (CD45RO, CXCR6, Granzyme B, Ki67) and RISH+ cells thereby defining cells as either positive or negative for these markers.

To define the hepatic lobule, lobular interface, portal interface and portal region, a pixel classifier was first trained to create a mask for Arginase+ hepatic lobule and then the mask (mask 1 = M1) was expanded 50 µm towards the hepatic lobule (mask 2 = M2) and 50 µm towards the portal region (mask 3 = M3). The M2 was defined as the hepatic lobule, the zone between M1 and M2 was defined as the lobular interface, the zone between M1 and M3 was defined as the portal interface and the zone between M3, and the liver explant mask was defined as portal region. Cells in the hepatic lobule (M1 zone) were visually and manually assigned into two classes as intrasinusoidal and extrasinusoidal. The density of RISH+ cells in each zone was calculated by dividing the total number of RISH+ cells in each zone to the area of the zone. All data were analyzed on raw pixel values and brightness and contrast were adjusted for visualization. Final figures were prepared with Fiji [11].

**Xenium In Situ**

The liver explant tissue was larger than the sample area in Xenium slide, therefore a region of interest (ROI) on the tissue block was determined based on the density of RISH+ cells in combined mIF/RISH staining described above. 5-µm thick section was obtained from the selected ROI in liver explant. The standard RNA quality control with DV200 and tissue morphology quality control with H&E staining were previously performed before TCRβ-CDR3 sequencing; therefore, these steps were not performed again before the Xenium in Situ experiment. Samples were processed at the Functional Genomics Center Zurich using Xenium v1 chemistry for FFPE samples with multimodal cell segmentation, following the manufacturer’s instructions. Briefly, samples underwent xylene-based deparaffinization before being transferred to Xenium cassettes. Decrosslinking was then performed, followed by an 18-hour probe hybridization step (for details on the gene panel and TCR clonotype probes, see the section “Design of add-on custom gene panel and TCR clonotype probes”). The following day, probe ligation and rolling-circle amplification were carried out. Subsequently, the samples were blocked and subjected to an 18-hour staining procedure with multimodal cell segmentation mix. This mix includes antibodies for staining cell membranes and cell interiors and a universal interior label for ribosomal RNA. On the next day, autofluorescence quenching and nuclear staining were performed. Finally, the processed Xenium slides were analyzed using the Xenium Analyzer. All experimental procedures strictly adhered to the protocols provided by 10x Genomics.

**Design of add-on custom gene panel and TCR clonotype probes for Xenium In Situ**

The 10x Genomics pre-designed Xenium Human Immuno-Oncology Profiling Panel (380 genes, Cat. No: 1000654) was supplemented with an additional genes (predesigned panel ID: VVWA4W, Cat. No: 1000651) chosen to characterize the inflamed liver and hyperexpanded T cell clones (for the complete gene list of add-on custom gene panel: **Supplementary Table 4**). Since the pre-design panel is missing the genes expressed in parenchymal cells of the liver, we added probes to identify transcripts highly expressed in hepatocytes (*HEPN1*, *GLUL*, *BCHE*, *CYP2A7*, *CYP3A7*) and cholangiocytes (*KRT7*, *KRT19*). The custom add-on panel also expanded the list of targeted transcripts which are expressed in immune and stromal cells such as T cells, NK cells, B-cells and plasma cells, stellate cells, endothelial cell, monocyte and macrophages. TCR probes were designed by the 10X Genomics Applied Bioinformatics team for CDR3 sequences corresponding to each shared hyperexpanded T cell clone identified in the liver explant (Cat. No: 1000664, for the complete TCR probe list, see **Supplementary Table 3**). Consequently, probes were successfully generated for three distinct hyperexpanded clones, with one probe per clone.

**Xenium In Situ Data Analysis**

Spatial transcriptomic data was generated using the 10x Genomics Xenium platform (instrument software version 3.2.1.2, analysis version xenium-3.2.0.7). Analysis was performed with R version 4.4.2 using Seurat v5 as the primary analytical framework. The images were obtained with Xenium Explorer version 3.2.0

**Cell segmentation and cell type annotation of Xenium In Situ**

Cell segmentation was performed based on multimodal cell segmentation immunofluorescence stain with standard Xenium segmentation algorithm provided by 10X Genomics that targets nucleus (DAPI), membrane (ATP1A1, E-Cadherin, CD45, 18S Ribosomal RNA) and cell interior (18S Ribosomal RNA, alphaSMA, Vimentin). T cell and NK cell populations were identified using scGate [12] with predefined models for NK cells (KLRD1+, CD3D-) and CD8+ T cells (CD8A+, CD3E+, KLRD1-). Dimensionality reduction with UMAP was used to visualize these populations, and feature expression was examined with density plots to confirm cell identity. Marker gene enrichment was validated through differential expression analysis between positive and negative populations using the Wilcox test (minimum percentage expression 10%, log fold change threshold 0.25). Cell types were classified by integrating graph-based clustering (resolution 0.4) with scGate results for T and NK cells. This approach generated a comprehensive annotation including Hepatocytes, Macrophages, General T cells, CD8 T cells, NK cells, Fibroblasts, Cholangiocytes, Monocytes, Endothelial cells, and B cells. Cell type identities were validated through the expression of canonical marker genes.

**T cell clonotype analysis with Xenium In Situ**

For each of three clones (clone1, clone2, clone3), cells were classified as positive based on the detection of at least one UMI count. The distribution of each clone was examined, with particular attention to CD8+ T cells and NK cells identified by scGate. Summary statistics were calculated, including total positive cells, percentage of all cells expressing the clones, and the proportion of positive cells within specific immune populations. Combined analyses with clone1, clone2, clone3 were performed to characterize their shared features and distribution across cell types. Differential gene expression analysis was performed between TCR+ and TCR- CD8+ T cells for each clone individually and for combined groupings. Expression of activation markers (*GZMA*, *GZMB*, *GZMH*, *GZMK*, *CST7*) and residency markers (*CXCR6*, *CD69*, *ITGAE*, *KLRB1*) was examined using dot plots and violin plots to determine the functional status of TCR+ CD8+ T cells.

**Sanger Sequencing**

Genomic DNA isolated from FFPE sample was PCR amplified with AmpliTaq Gold™ 360 Master Mix (ThermoFischer, 4398881) to evaluate ERAP2 rs1363907 (chr5: 96252803 [GRCh37/hg19]) variant. The PCR product was visualized on the agarose gel, purified and sequenced with sanger cycle sequencing (Microsynth AG, Switzerland). Primers used for PCR amplification (5’-3’): ERAP2_F: GCAGGTGGAGGTTACAATGAG and ERAP2_R: AGGTTGGCAGAGAAATGCC.

**Statistical Analysis**

The chi-square test with Bonferroni correction was used to compare categorical data. A two-tailed p value < 0.05 was used to infer statistical significance. All graphs were generated using GraphPad Prism version 9.2.0 and R Studio version 4.1.2.


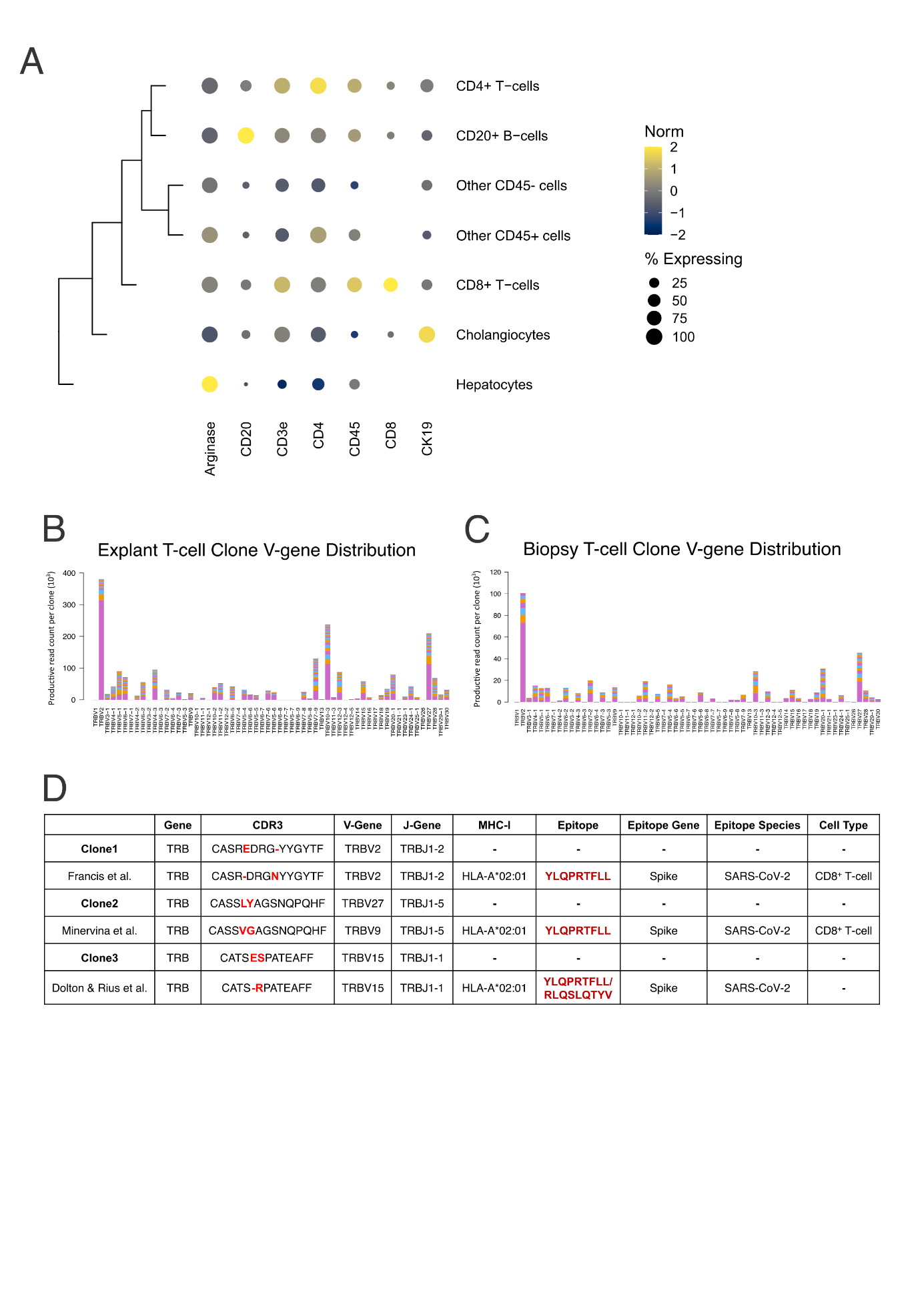


**Supplementary Figure 1. T cell immune repertoire of liver explant and biopsy of SVALI patient**

**A)** Dot-plot showing marker expression in each cluster. The size of the dot corresponds to the percentage of cells expressing the markers in each cluster. The color represents the scaled average expression level. **B)** The productive read counts per T cell clone in explant tissue. Each colour in stacked bars represents a different T cell clone. **C)** The productive read counts per T cell clone in liver biopsy. Each colour in stacked bars represents a different T cell clone. **D)** The three T cell clones that were shared between liver explant and liver biopsy and that were hyperexpanded in both liver biopsy and liver explant exhibited CDR3 amino acid sequence similarity to YLQPRTFLL-specific T cell clones.

**
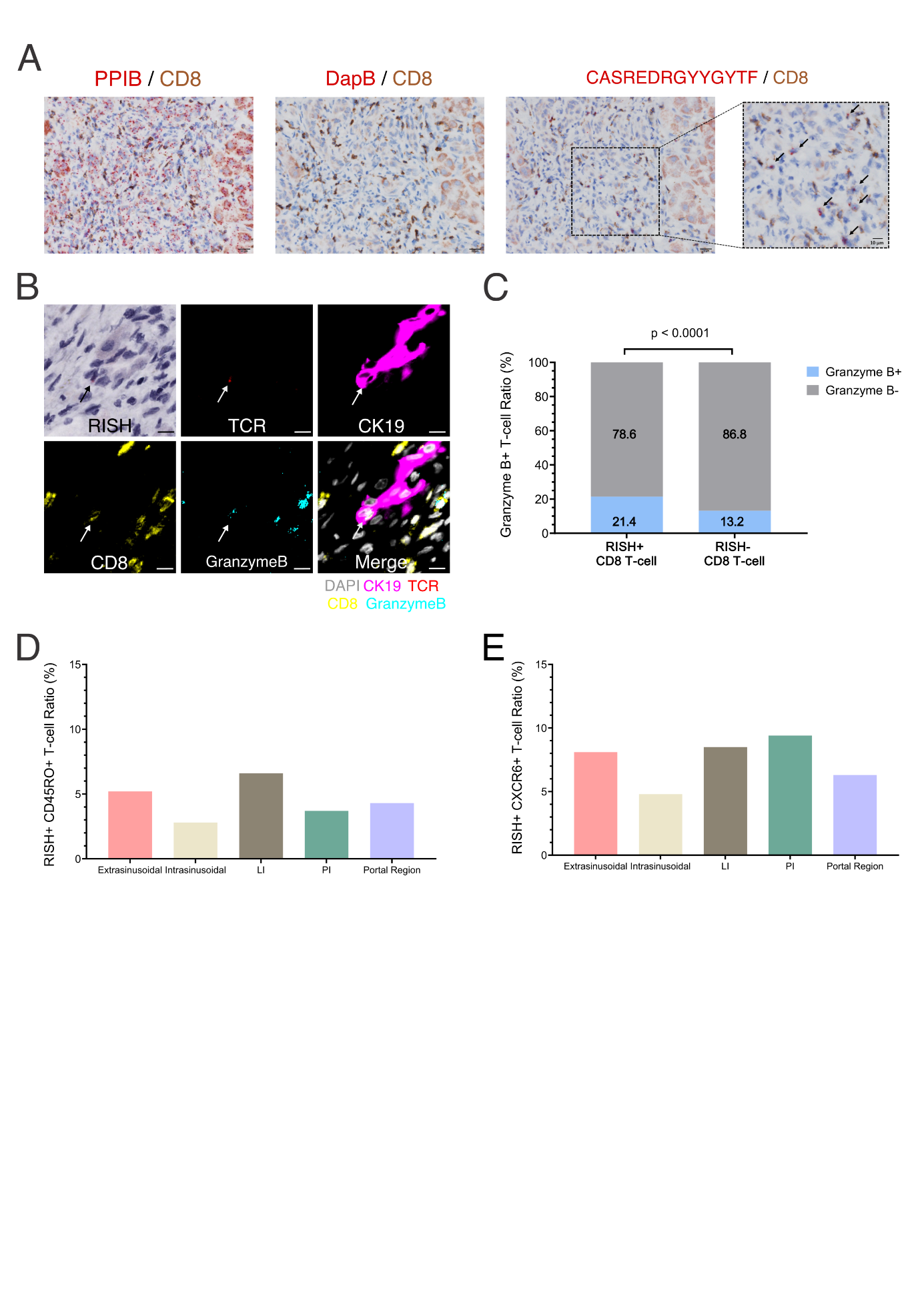
**

**Supplementary Figure 2. In situ localization and phenotyping of clone #1 in liver explant**

**A)** Characterization of RISH+ T cell clones with BaseScope dual RISH/IHC assay by using anti-CD8 antibody. Arrows show the CD8+ T cells that express the target CDR3 mRNA. **B)** Infiltration of bile ducts with Granzyme B+ CD8+ RISH+ T cells. The arrow shows RISH+ T cell clone. Merge image: DAPI= Gray, CK19= Magenta, CD8= yellow, Granzyme B= blue, TCR = Red. Scale Bar= 10 µm. **C)** Granzyme B+ T cell frequency among RISH+ CD8+ T cells and RISH- CD8+ T cells. **D)** The frequency of RISH+ CD8+ CXCR6+ T cells in different zones. **E)** The frequency of RISH+ CD8+ CD45RO+ T cells in different zones.**
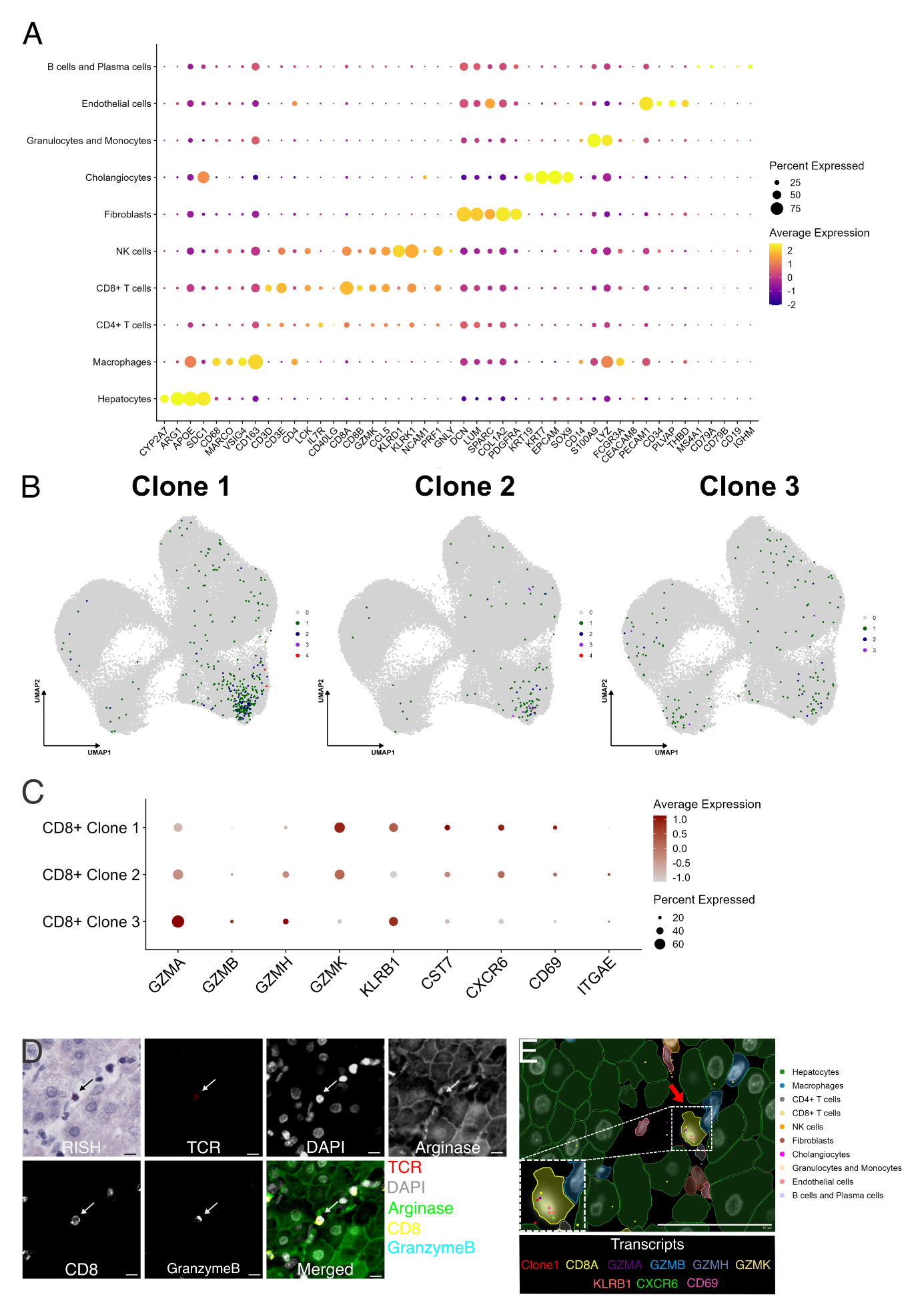
**

**Supplementary Figure 3. In situ localization and phenotyping of clone 1, 2 and 3 in liver explant with Xenium in situ**

**A)** Dot plot showing the feature expression of each cell phenotype. Dot size indicates the percentage of cells expressing indicated marker and colour bar shows the scaled average expression of the marker within a cell phenotype. **B)** UMAP projection of Xenium in situ data showing the number of TCR transcript per shared hyperexpanded T cell clone (clone1, clone2 and clone3). Each point represents a cell, and the colors indicate TCR transcript number per cell. **C)** Average expression of cytotoxicity and residency markers in TCR+ CD8+ Clone 1, Clone 2 and Clone 3. Dot size indicates the percentage of cells expressing indicated marker and colour bar shows the scaled average expression of the marker. **D)** Granzyme B+ CD8+ RISH+ T cells in hepatic sinusoid. The arrow shows RISH+ T cell clone. Merge image: DAPI= Gray, CK19= Magenta, CD8= yellow, Granzyme B= blue, TCR = Red. Scale Bar= 10 µm. **E)** Clone 1 (red arrow) in the hepatic sinusoid. Cell annotation masks indicate different cell types. Each dot represents an mRNA transcript detected by Xenium in situ, and mRNA transcripts are colour-coded, shown below the figure. Scale bar = 50 µm.

**
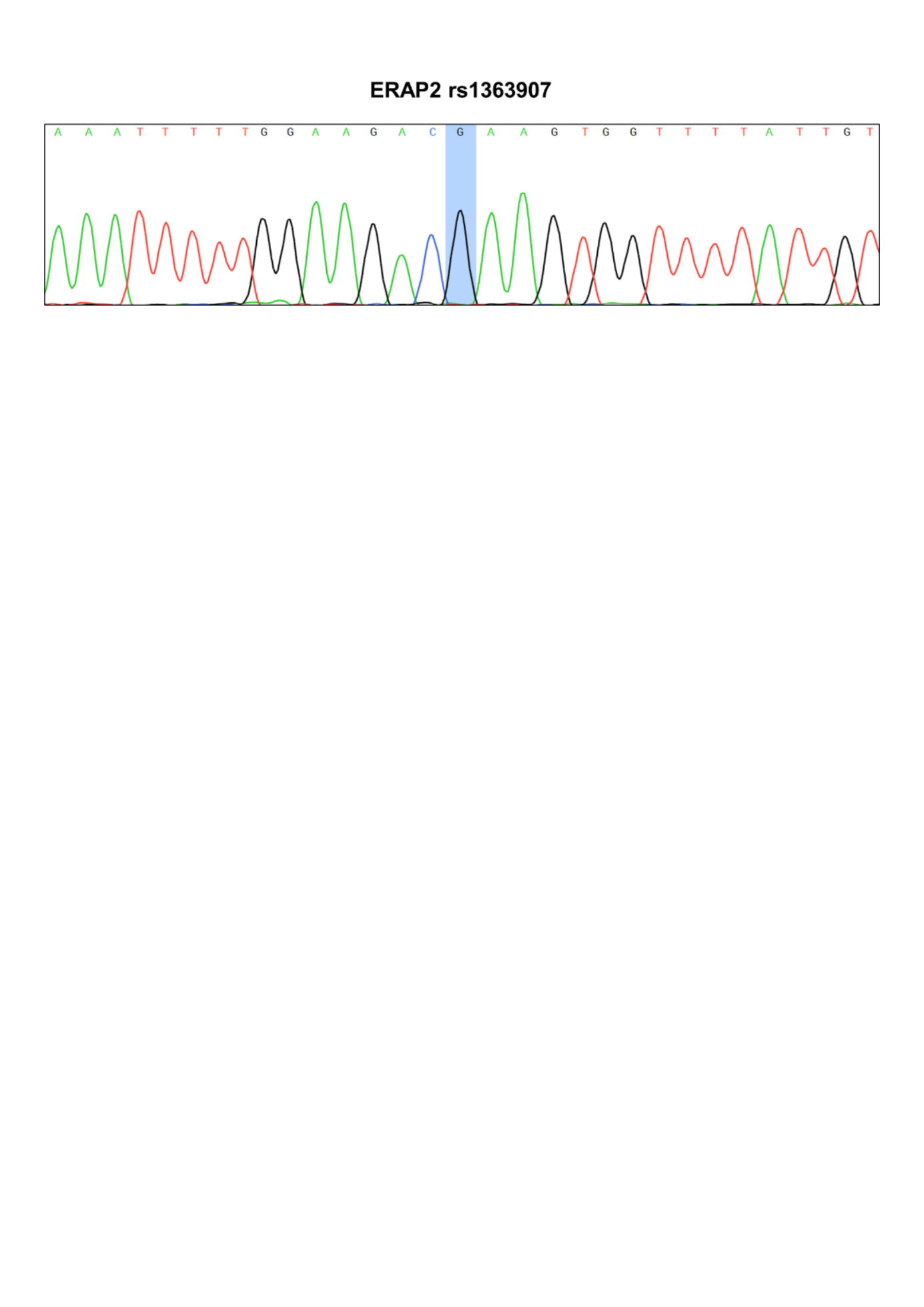
**

**Supplementary Figure 4.** The Sanger sequencing of the genomic region covering ERAP2 rs1363907

**Supplementary Table 1. T cell immune repertoire of liver explant and liver biopsy samples**

Supplementary Table 1 can be found as a separate supplementary document.

**Supplementary Table 2. Reagents used in Phenocycler-Fusion Experiment**

| **Primary Antibody** | **Company** | **Catalog Number** |
| --- | --- | --- |
| Arginase | Abcam | ab211961 |
| CD103 | Abcam | ab254201 |
| CD20 | Akoya Biosciences | 4450018 |
| CD31 | Akoya Biosciences | 4250009 |
| CD38 | Akoya Biosciences | 4250080 |
| CD3e | Akoya Biosciences | 4550125 |
| CD45 | Akoya Biosciences | 4250099 |
| CD45RO | Akoya Biosciences | 4250023 |
| CD4 | Akoya Biosciences | 4550112 |
| CD68 | Akoya Biosciences | 4550113 |
| CD69 | Biolegend | 310902 |
| CD8 | Akoya Biosciences | 4250012 |
| CK19 | Biolegend | 628502 |
| CXCR6 | Abcam | ab281934 |
| FoxP3 | Biolegend | 320002 |
| Granzyme B | Akoya Biosciences | 4250055 |
| HLA-DR | Akoya Biosciences | 4550118 |
| Interferon γ | Akoya Biosciences | 4250062 |
| Ki67 | Akoya Biosciences | 4250019 |
|  |  |  |
| **Reagents** | **Company** | **Catalog Number** |
| Flow Cell 10pk for PhenoCycler-Fusion | Akoya Biosciences | 240205 |
| 10X Buffer for PhenoCycler | Akoya Biosciences | 7000001 |
| Assay Reagent for PhenoCycler | Akoya Biosciences | 7000002 |
| Nuclear Stain for PhenoCycler | Akoya Biosciences | 7000003 |
| 96 well plates for PhenoCycler | Akoya Biosciences | 7000006 |
| 96 well plate seals for PhenoCycler | Akoya Biosciences | 7000007 |
| Staining Kit for PhenoCycler | Akoya Biosciences | 7000008 |
| Antibody Conjugation Kit | Akoya Biosciences | 7000009 |
| Amicon Ultra-0.5 Centrifugal Filter Unit | Sigma-Aldrich | UFC505024 |
| Paraformaldehyde 16% Aqueous Solution EM Grade | Electron Microscopy Sciences | 15710 |

**Supplementary Table 3. Probe sequences for hyperexpanded T cell clones**

| **Clones** | **rbd5-sequence** | **rbd3-sequence** | **Probe Length** |
| --- | --- | --- | --- |
| Clone1 | TGCCAGCAGAGAAGACA | GGGGATACTATGGCTAC | 34 |
| Clone2 | CCAGCAGTTTATACGCAGGG | AGCAATCAGCCCCAGCATTT | 40 |
| Clone3 | TGTGTGCCACCAGCGAG | AGCCCTGCCACTGAAGC | 34 |

**Supplementary Table 4. Add-on custom gene panel**

|  | **Gene** | **Ensembl ID** | **Number of Probes** |
| --- | --- | --- | --- |
| 1 | TNFRSF4 | ENSG00000186827 | 1 |
| 2 | SCD | ENSG00000099194 | 3 |
| 3 | KRT18 | ENSG00000111057 | 3 |
| 4 | PMEL | ENSG00000185664 | 3 |
| 5 | COL1A2 | ENSG00000164692 | 4 |
| 6 | TAGLN | ENSG00000149591 | 4 |
| 7 | BATF | ENSG00000156127 | 5 |
| 8 | LYZ | ENSG00000090382 | 5 |
| 9 | TRAV1-2 | ENSG00000256553 | 6 |
| 10 | TNFRSF13B | ENSG00000240505 | 7 |
| 11 | TNFSF8 | ENSG00000106952 | 8 |
| 12 | IFNG | ENSG00000111537 | 8 |
| 13 | CD69 | ENSG00000110848 | 8 |
| 14 | CCR8 | ENSG00000179934 | 8 |
| 15 | ICOS | ENSG00000163600 | 8 |
| 16 | IL21R | ENSG00000103522 | 8 |
| 17 | IL17A | ENSG00000112115 | 8 |
| 18 | B3GAT1 | ENSG00000109956 | 8 |
| 19 | BCL6 | ENSG00000113916 | 8 |
| 20 | AICDA | ENSG00000111732 | 8 |
| 21 | THBD | ENSG00000178726 | 8 |
| 22 | XCR1 | ENSG00000173578 | 8 |
| 23 | CLEC4C | ENSG00000198178 | 8 |
| 24 | CLEC9A | ENSG00000197992 | 8 |
| 25 | CD207 | ENSG00000116031 | 8 |
| 26 | HEPN1 | ENSG00000221932 | 8 |
| 27 | GLUL | ENSG00000135821 | 8 |
| 28 | CYP2A7 | ENSG00000198077 | 8 |
| 29 | CYP3A7 | ENSG00000160870 | 8 |
| 30 | BCHE | ENSG00000114200 | 8 |
| 31 | KRT7 | ENSG00000135480 | 8 |
| 32 | KRT19 | ENSG00000171345 | 8 |
| 33 | MUC1 | ENSG00000185499 | 8 |
| 34 | TTF1 | ENSG00000125482 | 8 |
| 35 | TP63 | ENSG00000073282 | 8 |
| 36 | DES | ENSG00000175084 | 8 |
| 37 | MYOD1 | ENSG00000129152 | 8 |
| 38 | MYOG | ENSG00000122180 | 8 |
| 39 | PAX7 | ENSG00000009709 | 8 |
| 40 | PAX3 | ENSG00000135903 | 8 |
| 41 | NECTIN3 | ENSG00000177707 | 8 |
| 42 | MLANA | ENSG00000120215 | 8 |
| 43 | SOX10 | ENSG00000100146 | 8 |
| 44 | CDH1 | ENSG00000039068 | 8 |
| 45 | LYVE1 | ENSG00000133800 | 8 |
| 46 | PDPN | ENSG00000162493 | 8 |
| 47 | PECAM1 | ENSG00000261371 | 8 |
| 48 | NCR1 | ENSG00000189430 | 8 |
| 49 | SIGLEC8 | ENSG00000105366 | 8 |
| 50 | FUT4 | ENSG00000196371 | 8 |
| 51 | MPO | ENSG00000005381 | 8 |
| 52 | CD5L | ENSG00000073754 | 8 |
| 53 | TRDC | ENSG00000211829 | 8 |
| 54 | CD7 | ENSG00000173762 | 8 |
| 55 | PDGFRB | ENSG00000113721 | 8 |
| 56 | CD34 | ENSG00000174059 | 8 |
| 57 | COL13A1 | ENSG00000197467 | 8 |
| 58 | COL11A1 | ENSG00000060718 | 8 |
| 59 | TOP2A | ENSG00000131747 | 8 |
| 60 | FAP | ENSG00000078098 | 8 |
| 61 | IL12A | ENSG00000168811 | 8 |
| 62 | BLK | ENSG00000136573 | 8 |
| 63 | CR2 | ENSG00000117322 | 8 |
| 64 | WDFY4 | ENSG00000128815 | 8 |
| 65 | FBLN2 | ENSG00000163520 | 8 |
| 66 | MMP14 | ENSG00000157227 | 8 |
| 67 | FABP5 | ENSG00000164687 | 8 |
| 68 | IL7 | ENSG00000104432 | 8 |
| 69 | S100A2 | ENSG00000196754 | 8 |

**Supplementary Table 5. Differentially expressed genes between TCR+ CD8+ cells and TCR- CD8+ cells**

|  | **Gene** | **avg_log2FC** | **p_val** | **p_val_adj** |
| --- | --- | --- | --- | --- |
| 1 | clone 1 | 19.62037939 | 0 | 0 |
| 2 | clone 2 | 17.23693999 | 2.08E-282 | 1.00E-279 |
| 3 | clone 3 | 17.1164617 | 1.93E-218 | 9.28E-216 |
| 4 | KLRB1 | 1.279730259 | 3.28E-30 | 1.57E-27 |
| 5 | SAMD3 | 0.698790254 | 2.53E-13 | 1.22E-10 |
| 6 | ATM | 0.671866702 | 2.14E-11 | 1.03E-08 |
| 7 | KLRF1 | 1.56681493 | 3.65E-11 | 1.75E-08 |
| 8 | TC2N | 0.547897048 | 5.05E-09 | 2.42E-06 |
| 9 | TRAT1 | 0.608489521 | 2.27E-06 | 0.001089071 |
| 10 | PTPRC | 0.465806892 | 9.29E-06 | 0.004461279 |
| 11 | TCF7 | 0.466183184 | 1.04E-05 | 0.004997815 |
| 12 | KLRK1 | 0.315928896 | 1.12E-05 | 0.005360875 |
| 13 | GZMK | 0.391257189 | 1.78E-05 | 0.008530089 |
| 14 | FGR | 0.768969914 | 2.31E-05 | 0.011067206 |
| 15 | CD2 | -0.522665642 | 2.88E-05 | 0.013826555 |
| 16 | CXCL14 | 1.146504194 | 3.91E-05 | 0.018747037 |
| 17 | CST7 | 0.629560163 | 4.20E-05 | 0.020171308 |
| 18 | VSIG4 | 0.516197863 | 7.54E-05 | 0.036196682 |
| 19 | CORO1A | 0.402364789 | 7.87E-05 | 0.037757209 |
| 20 | S100A9 | 0.260221972 | 8.15E-05 | 0.039105653 |
| 21 | KLRD1 | 0.581028242 | 0.000100384 | 0.048184223 |
| 22 | DCN | 0.295166716 | 0.000103572 | 0.049714445 |

**Supplementary References**

1. Efe C, Harputluoğlu M, Soylu NK, Yilmaz S. Letter to the editor: Liver transplantation following severe acute respiratory syndrome‐coronavirus‐2 vaccination–induced liver failure. *Hepatology*. 2022;75:1669–1671.

2. Efe C, Kulkarni AV, Terziroli Beretta‐Piccoli B, et al. Liver injury after SARS‐CoV‐2 vaccination: Features of immune‐mediated hepatitis, role of corticosteroid therapy and outcome. *Hepatology*. 2022;76:1576–1586.

3. Peng K, Nowicki TS, Campbell K, et al. Rigorous benchmarking of T-cell receptor repertoire profiling methods for cancer RNA sequencing. *Brief Bioinform*. 2023;24:bbad220.

4. Hadley Wickham. ggplot2: Elegant Graphics for Data Analysis. Springer-Verlag New York; 2016. https://ggplot2.tidyverse.org.

5. Goncharov M, Bagaev D, Shcherbinin D, et al. VDJdb in the pandemic era: a compendium of T cell receptors specific for SARS-CoV-2. *Nat Methods*. 2022;19:1017–1019.

6. Bankhead P, Loughrey MB, Fernández JA, et al. QuPath: Open source software for digital pathology image analysis. *Sci Rep*. 2017;7:16878.

7. Chiaruttini N, Burri O, Haub P, Guiet R, Sordet-Dessimoz J, Seitz A. An Open-Source Whole Slide Image Registration Workﬂow at Cellular Precision Using Fiji, QuPath and Elastix. 2022;3.

8. Uhlén M, Fagerberg L, Hallström BM, et al. Proteomics. Tissue-based map of the human proteome. *Science*. 2015;347:1260419.

9. Schmidt U, Weigert M, Broaddus C, Myers G. Cell Detection with Star-Convex Polygons. In: Frangi AF, Schnabel JA, Davatzikos C, Alberola-López C, Fichtinger G, eds. Medical Image Computing and Computer Assisted Intervention – MICCAI 2018. Cham: Springer International Publishing; 2018. p. 265–273.

10. Levine JH, Simonds EF, Bendall SC, et al. Data-Driven Phenotypic Dissection of AML Reveals Progenitor-like Cells that Correlate with Prognosis. *Cell*. 2015;162:184–197.

11. Schindelin J, Arganda-Carreras I, Frise E, et al. Fiji: an open-source platform for biological-image analysis. *Nat Methods*. 2012;9:676–682.

12. Andreatta M, Berenstein AJ, Carmona SJ. scGate: marker-based purification of cell types from heterogeneous single-cell RNA-seq datasets. *Bioinformatics*. 2022;38:2642–2644.
